# The Influence of Acute Aerobic Exercise on Craving Degree for University Students with Mobile Phone Dependency: A Randomized Controlled Trial

**DOI:** 10.3390/ijerph19158983

**Published:** 2022-07-23

**Authors:** Guan Yang, Rulan Shangguan, Yuanyuan Ke, Songtao Wang

**Affiliations:** 1 School of Physical Education, South China University of Technology, Guangzhou 510641, China; rlshangguan@scut.edu.cn (R.S.); keyuanyuan@email.hbue.edu.cn (Y.K.); 2 School of Sport Economics and Manage, Hubei University of Economics, Xiangyang 430205, China; 3 School of Physical Education and Sports Science, South China Normal University, Guangzhou 510006, China

**Keywords:** mental health, mobile phone dependency, acute moderate-intensity aerobic exercise, craving degree, visual analog scale, randomized controlled trial, university students

## Abstract

These days, mobile phone dependency (MPD) has become one of the most imperative and impressive puzzles in the area of behavioral addictions and public health across the world, especially the individuals with MPD that might frequently crave using mobile phones themselves. The target of the current study was to determine whether moderate-intensity aerobic exercise could reduce the craving degree for mobile phones for university students with MPD by a randomized, controlled trial. Sixty Chinese undergraduates, including 30 male and 30 female students aged from 18 to 22 years (20.08 ± 1.94 years) with MPD were recruited and then randomly assigned to the exercise group (*n* = 30) or the control group (*n* = 30) with even numbers by gender and major. Participants in the exercise group were required to perform an acute moderate-intensity treadmill exercise lasting for 30 min at 45–68% heart rate reserve (HRR) with background music, while the control group were only asked to listen to the same music for 40 min without any exercise. Sport watches were employed to monitor their heart rate (HR), and the exercise group was also obliged to report their rating of perceived exertion (RPE). After completing the experimental task, a visual analog scale (VAS) was used to evaluate their craving degree for mobile phones, and an independent samples *t*-test was computed to reveal the difference in the scores of craving degree for mobile phones between the two groups. The results showed that the VAS score for the exercise group (3.77 ± 1.36) was significantly lower (*p* < 0.001) than that of the control group (6.11 ± 1.39). The findings suggested that acute moderate-intensity aerobic exercise could be an effective and reliable means to help deal with the issue of mobile phones craving for the undergraduates with MPD, and more longitudinal intervention studies and control trial designs should be conducted in the near future to further test the long-term effects of this exercise.

## 1. Introduction

In recent years, mobile phones, especially smartphones, have been playing an increasingly indispensable role in our day-to-day lives [1,2]. In terms of interpersonal communication, public transportation, online shopping, instant payments, online courses, and information distribution, or even in aspects of watching videos, playing games, browsing newspapers, this tool can provide more and more convenience for us now [3,4]. Meanwhile, lifestyles and behavioral manners among humans have also been greatly changed by the intelligent features of this device. According to a recent report, the number of mobile phone users all over the world will exceed 4 billion by 2023 [5]. It is no exaggeration to say that, mobile phones are important to the masses as much as water to fish in this intelligent age.

However, it is unfortunate that an increasing number of people have become addicted to mobile phones, and especially that they are not inhibiting their usage of this advanced implement, which frequently causes a series of psychological and behavioral problems [1,4,6]. What is worse, the phenomena of problematic and pathological mobile phone usage can be seen anytime and anywhere [6,7], which in some individuals leads to the worst outcomes: mobile phone dependency (MPD) or mobile phone addiction (MPA) [8,9]. Chóliz, an expert from the area of mental health, predicted 12 years ago that MPD would become one of the most crucial behavioral addictions in the 21st century [8]. Xiong et al. has lately pointed out that the overall prevalence of MPD has reached 28.3% [10], which means that almost one out of every three people struggles with indulging in mobile phones.

Without a doubt, a large amount of research has gradually revealed that for residents across the world, MPD has not only brought lots of passive and negative effects on their physical and psychological health, such as anxiety, depression, insomnia, loneliness, and other similar awful symptoms, but also on their work routine and interpersonal communication [9,11,12,13,14]. In addition to the aforementioned risks, in students MPD has also jeopardized their academic performance [15,16]. According to previous work on drug addiction and other similar conditions, including Internet addiction and Internet game disorders [17,18,19], the reason why individuals cannot control mobile phone usage may be that they actually do not have the ability to inhibit these compulsive and impulsive behaviors; accurately speaking, they fail to restrain the craving to use their mobile phones, which means their cognitive function has been damaged to a large extent.

In a population with drug dependency, Wang and Zhu put forward that craving degree would be a valid indicator to evaluate incidence of relapse during withdrawal [20]. Meanwhile, Wang also argued that drug addicts’ weak inhibitory control abilities may be the principal reason causing drug abuse and relapse [21], suggesting that enhancing the inhibitory control ability for those people would be an effective and feasible means to ameliorate their caving degree for addictive substances [22,23]. Similarly to the addicts mentioned above, Zu found that the individuals addicted to mobile phones also have a poor inhibitory control ability, so that they usually fail to inhibit their craving for employing mobile phones [24]. At the same time, several prior survey studies also disclosed that there is a positive relationship between self-control and MPD [25,26]. According to these relevant studies above, it is not difficult to infer that if the people’s craving degree for mobile phones could be reduced or ameliorated, their compulsive or impulsive behaviors would directly decrease, and then their MPD could be also naturally attenuated to a large degree. Therefore, reducing craving degree on relevant addictive substance through some conducive and practicable ways might be a highly promising solution for the population of addicts.

As we all know, nowadays, the notion that exercise is an imperative and beneficial approach to maintain physical and psychological health in routine lifestyle has been widely accepted and practiced across the world [27]. In particular, the chronic and regular aerobic exercise recommended by the American College of Sports Medicine (ACSM) and the World Health Organization (WHO) would be the optimal means for the majority of people, such as youths, adults, the elderly, and even some clinical populations to promote health and prevent diseases [28,29,30,31]. More importantly, several published articles have also proved that aerobic exercise can be an effective and reliable means to help those who are dependent on heroine, nicotine, alcohol, and cigarettes recover by means of decreasing the craving degree for these addictive substance [21,32,33]. Simultaneously, the previous studies have also shown that appropriate physical activity, especially moderate-intensity aerobic exercise, could efficiently improve individuals’ self-control ability or inhibitory control ability [34,35,36], which implies that this exercise would be conducive to help the population indulging in mobile phones reduce their craving degree upon exposure of triggers relevant to this digital product by enhancing and intensifying their executive function. Similarly, a recent cross-sectional survey has also revealed an optimal dose−response relationship between physical activity and MPD; that is, moderate-intensity exercise would be the best intervention [37].

### The Present Study

In view of those analyses and discussions mentioned above, it is not difficult for us to infer that aerobic exercise can be a potential way to reduce the craving degree for mobile phones. Hence, the aim of the present study was to examine whether acute moderate-intensity aerobic exercise could have a positive impact on craving degree for mobile phones for individuals with MPD by a randomized, controlled trial. Furthermore, it is also hypothesized that compared to the subjects in the control group, the craving degree for mobile phones for university students with MPD in the exercise group would be significantly lower after performing a 30 min moderate-intensity aerobic exercise.

## 2. Materials and Methods

### 2.1. Participants

A single-factor between-subjects design was adopted in the current study, and 60 undergraduates, including 30 male and 30 female students aged from 18 to 22 years in China from a Southern Vocational University who were eligible and screened for relevant experimental requirements were enrolled in this study. The main criteria for eligibility were as follows: (1) meeting the diagnosis criterion of MPD tested by the standard scale, namely a score of MPD equal to or over 57; (2) without any inborn physical or mental conditions, that is, being both physically and mentally healthy; (3) having the ability to do moderate-intensity or moderate- to vigorous-intensity physical activity for at least 30 min. Then, the subjects were randomly and evenly assigned to the exercise group (*n* = 30) or the control group (*n* = 30), and each group included 15 female students and 15 male students, as well as 15 students who majored in liberal arts and 15 students who majored in science. This randomized, controlled trial was performed from November to December in 2020. In addition, each subject received RMB 50 Yuan as a bonus for their participation.

The present study was conducted in accordance with the Declaration of Helsinki, and was also approved by the Ethics Committee of the School of Physical Education and Sports Sciences at South China Normal University (No. SCNU-SPT-2020-001). Written informed consent was obtained from all subjects involved in this study, and they were all completely voluntary and anonymous throughout the entire study. The subjects also had the right to withdraw from this experiment at any time.

### 2.2. Measures

#### 2.2.1. Mobile Phone Addiction Tendency Scale

To screen the eligible participants, the Mobile Phone Addiction Tendency Scale for College Students (MPATS) was used to assess the degree of MPD [38]. This scale consisted of 16 items, such as “I prefer to choose a mobile phone to talk with others rather than a direct face-to-face communication”, and the answer will be chosen on a scale from totally disagree to totally agree. Each item was rated on a five-point Likert scale, with the total scores ranging from 16 to 80. A participant with a total score of 57 or above would be considered to be a subject having MPD [39]. The present scale has been demonstrated well to evaluate Chinese undergraduates with convincing reliability and validity [25,37].

#### 2.2.2. Physical Activity Rating Scale-3

Exercise volume for participants in daily life was assessed by the Physical Activity Rating Scale-3 (PARQ-3) [40]. This scale comprised three items, namely exercise intensity, exercise duration, and exercise frequency, and each item was rated on a five-point Likert scale. For example, the following question was asked: “How much time do you usually spend on exercise everytime?” The answer was selected from five items, namely less than 10 min, 11 to 20 min, 21 to 30 min, 31 to 59 min, and equal or more than 60 min. Exercise volume was evaluated by the formula below: intensity × (duration − 1) × frequency, with the total scores ranging from 0 to 100. This scale has been fairly verified as a reliable and valid instrument to measure an individual’s physical activity in China [41,42].

#### 2.2.3. Visual Analog Scale

The craving degree for mobile phone for subjects with MPD was evaluated by the Visual Analog Scale (VAS), which is a widely employed instrument to accurately and objectively assess addicts’ craving degree for specific addictive materials [20,33,36], including mobile phones [24]. The VAS is a ten-point Likert self-reported scale made by the research team according to the corresponding standard and requirement, with 1 indicating a very weak craving degree for mobile phones and 10 a very strong craving degree [20,21]. In the present study, from 1 to 10 presented a gradually increasing craving degree for mobile phone by the subjects with MPD. That is, a larger number indicated a higher craving degree for mobile phones.

#### 2.2.4. Rating of Perceived Exertion Scale

In addition to sports watches, ratings of perceived exertion (RPE) were also used to monitor the exercise intensity of the subjects during the treadmill exercise, so that the accuracy of exercise intensity can be further enhanced. Therefore, the RPE6-20 scale was utilized in this study, in which RPE-6 means the resting heart rate (HR) was about 60 times per min, and RPE-20 means the maximum HR during exercise was approximately 200 times per min [32]. That is, from RPE-6 to RPE-20, the individual needed to gradually make more effort to maintain the present exercise condition. Actually, this scale has been broadly accepted by the American College of Sports Medicine (ACSM) [31] and ubiquitously employed by relevant researchers all over the world [43], due to the fact that it can effectively and accurately measure individuals’ exercise intensity.

#### 2.2.5. Demographic Variable

Several relevant and important demographic variables, including age, gender, major, height, and weight, were obtained from participants in a standard questionnaire. The body mass index (BMI) was calculated in accordance with the formula as follows: BMI = weight/height^2^.

### 2.3. Experimental Tasks

#### 2.3.1. Acute Aerobic Exercise Intervention

The aerobic exercise was divided into three parts, including a 5 min warm-up, a 30 min moderate-intensity treadmill exercise, and a 5 min cool-down. Specifically, exercise intensity was adjusted by changing the speed of the treadmill, so that subjects’ HR stayed within the range of 45–68% heart rate reserve (HRR) throughout the 30 min exercise [11,16]. The maximum HR was calculated by the formula as follows: 207 − 0.7 × age [30,44], and the HRR can be obtained via the formula: maximum HR minus resting HR. Thus, the target intensity during the aerobic exercise could be obtained as following: resting HR plus 45–68% HRR. In the entire period of the treadmill exercise, sport watches were employed to examine the condition of the subjects’ HR, and were worn on their left wrist. Meanwhile, the RPE was also required to be reported by them. Specifically, the subjects needed to report their current HR and level of RPE every 3 min, and these data were recorded by the researcher. If the HR was in the range of moderate-intensity aerobic exercise, they would only need to keep their present pace. If the HR was outside this range, the speed of the treadmill would be increased or reduced so as to keep their HR in the expected range. At the same time, a list of songs was automatically played by a portable music player to make sure all subjects were situated in the same musical environment.

#### 2.3.2. Evaluation of Craving Degree for Mobile Phone

To precisely evaluate the subjects’ craving degree for mobile phones, some mobile phone-relevant triggers, such as different mobile phone types, mobile phone wallpapers, mobile phone applications, mobile phone ringtones, mobile phone games, mobile phone movies, and other images of mobile phones, were collected and used as a pool, from which ten pictures were randomly selected and presented in slides on a screen. These various forms of triggers were randomly displayed to the participants at a speed of 1 page per second [24,32]. Upon the completion of the slideshows, the VAS was used to measure their craving degree for mobile phones; in other words, by asking the subjects to report or point to a specific number on the scale that represented their craving degree for mobile phone at that moment. Then this value would be immediately recorded by us.

### 2.4. Procedures

The present study was a single-factor design between groups (the exercise group vs. the control group), and 60 eligible subjects were randomly and evenly assigned to the exercise group and the control group to balance gender and major. Moreover, in order to ensure the homogeneity between the groups to obtain accurate and reliable results, the random assignment and baseline tests were performed prior to the formal treatment. The whole experiment was conducted from 8:30 to 11:30 in the morning, and each subject took a session lasting for almost an hour. The indoor temperature was controlled at about 25 degrees Celsius, and the humidity was controlled within the range of 50–60%.

For the exercise group, all subjects were required to perform an acute moderate-intensity treadmill exercise lasting for 30 min at 45–68% HRR while listening to music in the background. The specific procedures were as follows. At first, the resting HR would be calculated by us in order to compute the target HR during treadmill exercise. Secondly, in the warm-up, they walked slowly on the treadmill and then the speed of the treadmill was gradually increased, lasting for 5 min. Meanwhile, several songs prepared by us in advance were also played via a portable Apple tablet PC. Then, when the participants’ HR reached the lower interval of moderate-intensity aerobic exercise, they had to run on the treadmills, lasting for 30 min. In the period of treadmill exercise, their HR must be in the expected fluctuation range. After that, the speed of the treadmill was gradually reduced, and they began to run slowly on the treadmill until they could walk at a regular speed and relax themselves, which took nearly 5 min. At last, the music player was stopped, and the participants were required to conduct the evaluation of craving degree for mobile phones using the VAS.

For the control group, they only needed to listen to music for 40 min without any exercise. During this process, they also required to wear sports watches on their left wrists, and meanwhile, similar to the exercise group, their condition of HR was also recorded by us every 4 min. After that, they had to complete the evaluation of craving degree for mobile phones using the VAS. In addition, as a bonus, all subjects would receive RMB 50 for their participation.

### 2.5. Statistical Analysis

All statistical data in the current study were analyzed in SPSS version 26.0. Continuous variables were displayed as mean ± standard deviation (SD), and categorical variables were shown as frequency (*n*). The Independent samples *t*-test was employed to compare differences between the exercise group and the control group, and the significance level was set as 5%.

## 3. Results

### 3.1. Demographic Characteristics

All demographic information is presented in Table 1. The independent samples *t*-test indicated there were no significant differences in age, height, weight, BMI, MPD, and exercise volume between the exercise group and the control group. The baseline data revealed that there was no significant difference between the two groups.

### 3.2. Manipulation Check

During the formal procedure, the data of HR and RPE for the exercise group and HR for the control group were recorded every 3 min for a total of 10 times, and the averages are presented in Table 2. As for the exercise group, the average HR was (145.77 ± 5.69) times per min which is significantly higher than the average HR, (72.63 ± 3.26) times per min, for the control group (*t*_(58)_ = 61.05, *p* < 0.001, *d* = 15.76). Meanwhile, the average score of RPE reported by the exercise group was 12.63 ± 0.93, which means the participants felt it was somewhat hard to keep up the treadmill exercise. These results above showed that the subjects in the exercise group reached the expected exercise load, namely the moderate-intensity aerobic exercise.

### 3.3. Craving Degree for Mobile Phone

**Figure 1:**
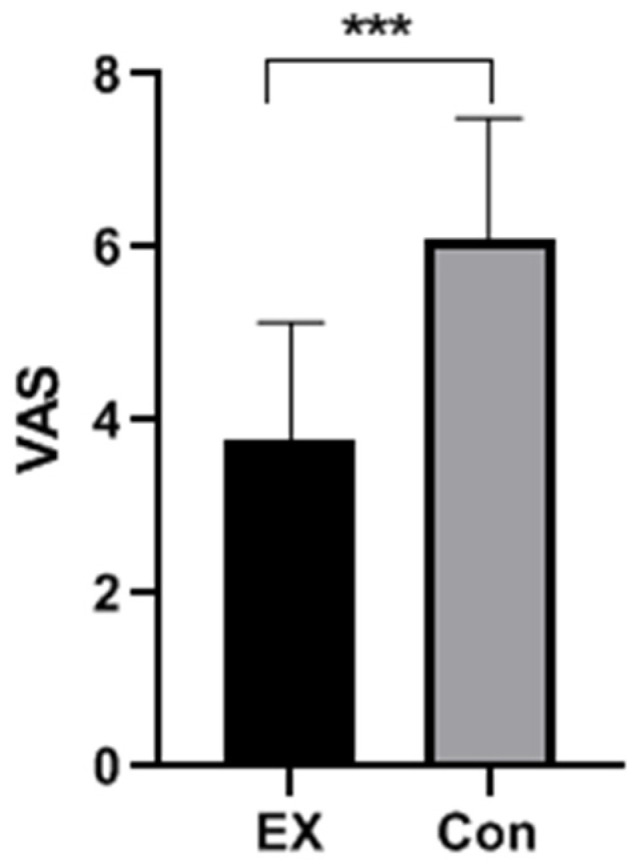
The score of VAS on evaluating subjects’ craving degree for mobile phones. Note: Ex: the exercise group; Con: the control group; VAS: visual analog scale. *** *p* < 0.001.

As clearly shown in Figure 1, the independent samples *t*-test indicated that upon completion of the acute moderate-intensity aerobic exercise, the exercise group had a significantly lower (*t*_(58)_ = 6.60, *p* < 0.001, *d* = 1.69) craving degree for mobile phones measured as the VAS score (3.77 ± 1.36) than the control group (6.12 ± 1.39).

## 4. Discussion

At present, it is known to us that MPD has become one of the most imperative and impressive puzzles in the area of behavioral addiction and public health across the world, especially as those addicted to mobile phones frequently do not inhibit their cravings for using these advanced electronic products. Likewise, based on the prior literature with respect to drug dependence and other behavioral addictions, the addicts may constantly fail to restrain their desire for those addictive substances, which has been universally viewed as the principal reason that hinders them from getting rid of this dilemma. Fortunately, aerobic exercise with proper intensity has been verified as a feasible and useful avenue to cope with this issue by means of attenuating their craving degree for these addictive substances. Given that, the target of the current study was to examine whether moderate-intensity aerobic exercise could reduce the craving degree for mobile phones for university students with MPD, and the hypothesis that 30 min of moderate-intensity aerobic exercise might significantly decrease the craving degree for mobile phones for university students with MPD has been supported by the present study.

Simultaneously, considering that gender and major might be the confounding factors between the groups as reported in previous research [41,45,46], this study also balanced this variable by using an even allocation to minimize the potential error. The results showed that the average HR for participants in the exercise group had reached the expected target, namely 45–68% HRR, and was significantly higher than those in the control group. Furthermore, the average value of RPE reported by the subjects in the exercise group was also corroborated by the change in HR for them maintained in the fluctuation range of moderate-intensity aerobic exercise. Additionally, the score of VAS on the craving degree for the exercise group was significantly lower than the score of the control group. Therefore, the assumption of the present study was fairly confirmed; that is, acute moderate-intensity aerobic exercise can effectively ameliorate the craving degree for mobile phones for undergraduates with MPD. To our knowledge, this is the first work to investigate whether the acute moderate-intensity aerobic exercise could bring positive and beneficial influences, namely decreasing craving degree for mobile phones, for university students addicted to smartphones.

Consistent with previous studies about drug dependence, the present study also found that this exercise can also ameliorate the craving degree for mobile phones of university students in China. For example, through the implementation of a program of moderate-intensity aerobic exercise that lasted for 12 weeks, Wang and Zhu disclosed that methamphetamine-dependent individuals’ craving degree was significantly reduced, but such improvement was not found in the control group; specifically, starting from the the third week, there was a significant decrease in craving degree for subjects in the experimental group [20]. Given the results from Wang and Zhu, it is also reasonable to conclude that the longer the intervention is conducted, the more effective the intervention might be [20]. Likewise, for those dependent on nicotine, Zhou also found that after having moderate-intensity aerobic exercise on the treadmill for 20 min, the individuals reported significantly lower scores of VAS than those in the control group [32]. Similarly, Taylor et al. recruited 20 alcohol-dependent people, and then conducted a within-group interaction design. They finally revealed that, compared to sitting silently for 15 min, brisk walking for the same amount of time led to a significant decrease in the score of VAS [47]. Moreover, in a study that recruited 20 opiate-dependent subjects, Bailey and Hall discovered that a 20 minute exercise led to more than 50% deduction in their VAS scores comparing to the control group, regardless of whether it was at the end of the exercise or just 10 min after the start of the exercise, the craving degrees for the treatment group tested by the VAS all decreased more than 50% [48].

Fatseas et al. put forth that craving, as a psychological urge when the addicts want to reacquire addictive behaviors, has been viewed as the core characteristic that maintains the passivity, and intensifies the addiction [49]. In addition, triggers relevant to addiction may be the main cause of cravings [50]. Fortunately, accumulated evidence has shown that whether through chronic aerobic exercise or acute aerobic exercise, the craving degree for different drug addicts can be ameliorated. More importantly, this positive effect has also emerged in the individuals addicted to mobile phones in the present work. At the same time, it is reasonable to discern that avoiding exposure to triggers relevant to mobile phones for those with MPD would be a highly efficient implementation to attenuate their craving degree, and their compulsive or impulsive behaviors on using mobile phones could also be decreased by a large degree.

Why can aerobic exercise be successfully applied to attenuate or reduce craving degree for mobile phones for university students with MPD? This puzzle can be clearly discerned from the published literature on drug dependence and behavioral addictions. Specifically speaking, the key point might be that exercise can enhance their cognitive control, namely inhibitory control or self-control, so that their ability to inhibit cravings for these addictive substances can be greatly reinforced, ultimately reducing their compulsive and impulsive behaviors in daily life [19,22,23]. In light of an extensively accepted model of addiction theory [51,52], an addict’s core brain areas related to cognitive control, such as the hippocampus, the nucleus accumbent, the orbitofrontal cortex, the anterior cingulate, and the prefrontal cortex, have been grievously impaired in the course of repeatedly using addictive substances, and serious disorders have gradually emerged in their inhibitory control ability. In particular, when addicts are exposed to contexts that are full of many substances relevant to addictive triggers, they may frequently fail to control themselves to inhibit cravings for those substances [53]. However, it is fortunate that aerobic exercise can repair impaired cognitive function and increase inhibitory control ability, so as to maximally reduce the craving degree or abuse of these addictive substances [54,55]. For example, for addicts dependent on marijuana or opium, Roessler conducted an aerobic exercise study lasting for 6 months and revealed that their self-control abilities were greatly boosted, and there was a significant reduction in craving degree for these substances [56].

On the basis of the published research [57,58,59], addicts usually do not have adequate self-control, and therefore are not able to inhibit their compulsive or impulsive behaviors to seek out those addictive substances; those with MPD are no exception. Similar to those with other behavioral addictions, individuals addicted to mobile phones may not control their time and behaviors in using this digital product, known as problematic or pathological mobile phone usage [1,60]. Moreover, several prior survey studies also revealed an obviously negative correlation between MPD and self-control [25,26]. According to the energy model of self-control proposed originally by Baumeister and his colleagues [61,62,63], self-control has been regarded as a limited source. For those who want to restrict or overcome their compulsive or impulsive behavior, this source would be consumed rapidly, resulting then in ego-depletion. Without a doubt, those with poor self-control and especially ego-depletion are more prone to compulsive or impulsive mobile phone usage. In addition, previous studies have also showed that the impulsive pathway could be one of the three most important symbols of problematic mobile phone use [64,65]. Nevertheless, it is fortunate that physical exercise, especially aerobic exercise, has been proved to be an imperative and practicable approach to enhance individuals’ self-control [66,67,68]. It can be seen that aerobic exercise can ameliorate the craving degree for mobile phones via strengthened self-control to inhibit the compulsive or impulsive usage of mobile phones.

Without a doubt, no matter reducing the craving degree for mobile phones directly through physical exercise per se, or indirectly through the improved self-control resulted by physical exercise, it is reasonable to infer that the key is to enhance the individual’s level of physical exercise in daily life. That being said, how can we obtain this? Luckily, recent high-quality research has highlighted that specifically, intrinsic motivation is related to an individual’s daily physical activity [69]; moreover, an individual’s intrinsic motivation towards physical exercise can be improved by providing them autonomy, competence, and related support [70]. Therefore, in accordance with the study mentioned above, this approach would be an effective and practical way to help those with MPD to improve their physical activity level, and then this troublesome issue could be gradually solved. To sum up, it is reasonable to conclude from this work that acute moderate-intensity aerobic exercise could be an effective and reliable means to help us deal with the issue of mobile phone cravings in the undergraduates with MPD. Consequently, for university students, several regular exercise programs, such as running, swimming, cycling, jogging, and tai chi, would be effective approaches to implement physical exercise in their daily life. Meanwhile, we also need to pay special attention to the exercise load; it should stay in the moderate-intensity range during exercise. Additionally, it must be put forward that in the future, a longitudinal intervention study and more control trial designs should be conducted to further testify the reliability of this result from the current research, as well as the long-term effects of moderate-intensity aerobic exercise.

Despite these valuable findings, there are also some limitations in the present study. First, this study only adopted acute aerobic exercise; hence, chronic aerobic exercise lasting for at least 8 weeks should be carried out to examine these positive benefits in the future. Secondly, the participants only included Chinese undergraduates, so studies that recruit university students from other countries are needed to strengthen the generalization. In addition, selecting other populations domestically or abroad, instead of university students, to further prove the outcome of the current study may be also indispensable and meaningful in the future. Fourthly, looking at relevant psychological or behavioral trials, the inhibitory control, cognitive control, and self-control of individuals with MPD should also be examined for more underlying findings. Furthermore, more advanced and objective instruments should be developed and applied for the purpose of conducting a more valid evaluation of instant psychological changes, such as the monitoring of the neural mechanisms through functional magnetic resonance image (fMRI) or event-related potentials (ERP) techniques. Last but not least, only a subjective measure of participants’ craving degree for mobile phones was applied in this study, yet an indirect behavior measurement, namely the so-called attention bias test, could be utilized to assess craving degree for mobile phones in the near future.

## 5. Conclusions

Compared to individuals from the control group, significantly lower craving degrees for mobile phones were observed among the subjects in the exercise group after performing a 30 min moderate-intensity aerobic exercise on the treadmills, suggesting that acute moderate-intensity aerobic exercise would be a very beneficial and practicable pathway to help efficiently control the craving degree for mobile phones in university students with MPD.

## Figures and Tables

**Table 1:** Demographic characteristics of subjects.

Variable	Type	Ex (*n* = 30)	Con (*n* = 30)	Total (*n* = 60)
Age	——	19.50 ± 1.74	19.67 ± 1.99	20.08 ± 1.94
Height (m)	——	1.65 ± 0.08	1.65 ± 0.09	1.65 ± 0.08
Weight (kg)	——	52.77 ± 8.31	55.82 ± 8.07	54.19 ± 8.51
BMI (kg/m^2^)	——	19.29 ± 1.96	21.11 ± 2.09	20.48 ± 2.21
MPD	——	61.23 ± 5.14	60.60 ± 3.62	60.92 ± 4.42
Exercise Volume	——	16.17 ± 10.23	15.73 ± 10.51	15.95 ± 10.38

Note: Ex: exercise group; Con: control group; MPD: score of MPATS; Exercise Volume: score of PARQ-3; BMI = weight/height^2^.

**Table 2:** Exercise intensity evaluation of subjects.

Variable	Exercise Group	Control Group
HR (t/min)	145.77 ± 5.69 ***	72.63 ± 3.26
RPE	12.63 ± 0.93	——

Note: RPE: rating of perceived exertion. *** *p* < 0.001.

## Data Availability

The data of the present experimental study can be available from the corresponding author via the reasonable request.
